# Investigation of the Emerging Nosocomial *Wickerhamomyces anomalus* Infections at a Chinese Tertiary Teaching Hospital and a Systemic Review: Clinical Manifestations, Risk Factors, Treatment, Outcomes, and Anti-fungal Susceptibility

**DOI:** 10.3389/fmicb.2021.744502

**Published:** 2021-10-06

**Authors:** Li Zhang, Meng Xiao, Amir Arastehfar, Macit Ilkit, Jun Zou, Yuchen Deng, Yingchun Xu, Wanqing Liao, Jingjun Zhao, Wenjie Fang, Weihua Pan

**Affiliations:** ^1^Department of Dermatology, Tongji Hospital, Tongji University School of Medicine, Shanghai, China; ^2^Peking Union Medical College Hospital, Beijing, China; ^3^Center for Discovery and Innovation, Hackensack Meridian Health, Nutley, NJ, United States; ^4^Division of Mycology, Faculty of Medicine, Çukurova University, Adana, Turkey; ^5^The Fourth People’s Hospital of Nanning, Nanning, China; ^6^Shanghai Key Laboratory of Molecular Medical Mycology, Department of Dermatology, Second Affiliated Hospital of Naval Medical University, Shanghai, China

**Keywords:** *Wickerhamomyces anomalus*, risk factors, treatment, outcomes, anti-fungal susceptibility, azoles, amphotericin B

## Abstract

*Wickerhamomyces anomalus* is an emerging pathogen, which has been associated with clonal outbreaks and poor clinical outcomes. Despite being an important emerging yeasts species, our understanding concerning the microbiological and clinical characteristics of infections due to this species is limited. Herein, we are reporting a retrospective analysis of fungemia patients with *W. anomalus* from a 2,100-bed hospital in Shanghai during 2014–2016. Moreover, we conducted an extensive literature review to gain a deeper clinical and microbiological insights. Detailed clinical data were recorded. Antifungal susceptibility testing (AFST) followed CLSI M27-A3, and isolates were identified using MALDI-TOF MS. In total, 13 patients were identified with a mortality rate of 38.5% (5/13). Central venous catheter (CVC), broad-spectrum antibiotic therapy, total parenteral nutrition (TPN), surgery, and mechanical ventilation were the most frequently observed risk factors. Eight patients (61.5%) experienced mixed bacterial/Candida bloodstream infections, and four patients developed mixed candidemia (MC). *W. anomalus* isolates showed high minimum inhibitory concentrations (MICs) against all azoles tested and flucytosine, while AMB showed the highest *in vitro* activity. Azoles were used for 84.6% (11/13) of the cases, while 36.4% (4/11) of them died. When combining with the AFST data and the literature review, our study highlights the poor efficacy of azoles and optimal efficacy of AMB and LAMB against infections caused by *W. anomalus*. In conclusion, our study highlights the emerging threat of *W. anomalus* affecting both neonates and adults. Furthermore, our results advocate the use of AMB formulations rather than azoles among patients infected with *W. anomalus*. Future studies are warranted to reach a definitive consensus regarding the utility of echinocandins among such patients.

## Introduction

Candidemia is a life-threatening infection caused by yeast species within the *Candida* genus, which can result in high morbidity, mortality, and extra hospital costs in the healthcare settings (Pappas et al., 2018). Five species of *Candida*, namely, *C. albicans*, *C. glabrata*, *C. tropicalis*, *C. parapsilosis*, and *C. krusei*, account for the vast majority of the candidemia cases (Wisplinghoff et al., 2004; McCarty and Pappas, 2016). *Candida albicans* is considered as the leading cause of nosocomial candidemia in most clinical settings; however, non-*Candida albicans* species, as well as several rare yeast species have been increasingly reported in clinical settings (Clancy and Nguyen, 2017; Fu et al., 2017).

*Wickerhamomyces anomalus* is an environmental yeast, mainly found in soil, plants, and fruit juices (Ma et al., 2000), and has been rarely isolated from clinical samples (Neumeister et al., 1992; Park et al., 2008; Ratcliffe et al., 2011). New lines of studies, however, have revealed its clinical importance and have implicated this species in a wide range of fungal infections, such as keratitis, meningitis, and candidemia, in immunocompromised and neonatal patients (Neumeister et al., 1992; Park et al., 2008; Ratcliffe et al., 2011). Moreover, this species has been associated with a relatively high mortality rate (41.2%) (Pasqualotto et al., 2005), and numerous studies have found this species as a cause of outbreaks (Kalenic et al., 2001; Kalkanci et al., 2010; Jung et al., 2018), especially among neonates (da Silva et al., 2013; Lin et al., 2013; Yang et al., 2021). While molecular tools failed to be identified from the hands of healthcare workers, they have revealed that *W. anomalus* isolates obtained from the outbreaks are genetically related, and importantly, the application of strict infection control and hand hygiene practices has resulted in eradication of such infections in hospitals with ongoing outbreaks due to *W. anomalus* (Pasqualotto et al., 2005). Moreover, *W. anomalus* isolates have an intrinsic high minimum inhibitory concentration (MIC) value to fluconazole, which is regarded as the most widely used antifungal drug used in developing countries (Arastehfar et al., 2019b, 2020).

According to the China Hospital Invasive Fungal Surveillance Net (CHIF-NET), *W. anomalus* becomes an emerging pathogen with incidence rising from 1.4% in 2009–2014 up to 2.5% of all isolates in 2015–2017 in China (Xiao et al., 2018, 2020). Evidently, such data point out the fact that fungemia due to *W. anomalus* are on the way of becoming more prevalent in clinical settings and also that *W. anomalus* is among the most important emerging yeast species potentially with high clinical implications.

Despite being an increasingly identified yeasts in clinical settings and being implicated in outbreaks, important clinical data, such as response to antifungal drugs and mortality, are still limited, which can potentially restrain our understanding of establishing an effective treatment option against infections caused by this species. Moreover, the microbiological data, especially antifungal susceptibility patterns as the cornerstone of clinical practices helping with establishing an effective therapeutic antifungal regimen, are limited. Additionally, access to antifungal susceptibility data can also aid in establishing epidemiological cut-off values (ECVs). As a result, clinical studies involving adequate number of patients suffering from fungemia due to *W. anomalus* would be an important step to close this knowledge gap.

Therefore, the scope of the current study was to systematically investigate 13 adult patients who suffered from *W. anomalus* fungemia in a Chinese tertiary teaching hospital, which was supplemented with a systemic review of the literatures, where adult patients with *W. anomalus* fungemia have been reported.

## Materials and Methods

### Study Design and Cases Collection

We retrospectively reviewed candidemia cases in Shanghai Changhai Hospital, a 2,100-bed hospital in Shanghai, China from January 2014 to July 2016. Candidemia was defined when ≥1 positive blood culture for *Candida* species were obtained. Patients with fungemia due to *W. anomalus* were included in this study.

The medical records of all *W. anomalus* infected patients were systematically reviewed. Baseline data were collected from the electronic medical records which included the patient’s gender and age, ward location, hospital stay, time to first isolate, underlying disease, mixed infections of other types of *Candida* species and bacteria, prior usage of broad-spectrum antibiotics or exposure to systemic antifungal agents and steroids 7 days before infections, mechanical ventilation, surgery, laboratory exanimations, antifungal therapy after infections, and outcomes. Total parenteral nutrition (TPN) and presence of central catheter [Central Venous Catheter (CVC) or Peripherally Inserted Central Catheter (PICC)] at the time were also collected.

### Identification of *Wickerhamomyces anomalus* and *in vitro* Antifungal Susceptibility Testing

All *Candida* isolates were identified by Vitek 2 Compact YST (BioMerieux, Lyon, France) and confirmed by matrix-assisted laser desorption ionization-time of flight mass spectrometry (MALDI-TOF MS) (Bruker Daltonics, Bremen, Germany) to avoid to be misidentified as *C. fabianii* and *C. utilis* (Arastehfar et al., 2019a). Antifungal susceptibility testing including five agents—flucytosine, amphotericin B, fluconazole, itraconazole, and voriconazole—was performed for each strain using the Clinical and Laboratory Standards Institute broth microdilution method (Rex et al., 2008) in Peking Union Medical College Hospital. MICs were determined by incubating at 35°C for 24 h. *C. krusei* ATCC 6258 and *C. parapsilosis* ATCC 22019 were included as quality controls in each antifungal susceptibility testing experiment. Susceptibility to antifungal drugs was assessed as suggested previously (Pfaller and Diekema, 2012). Strains with MIC values >4 and > 0.25 μg/ml were considered as non-wild type (NWT) to fluconazole and voriconazole, respectively. Since there was no ECVs and/or clinical breakpoints (CBPs), the MICs of flucytosine, itraconazole, and AMB reported were compared to those of *C. albicans*. Therefore, isolates displaying MIC values of >2, 0.5, and >0.125 μg/ml were noted as NWT against AMB, flucytosine, and itraconazole, respectively (Pfaller and Diekema, 2012). Data was analyzed using SPSS 25.

### Review of the Literature

We screened using the key words of *Wickerhamomyces anomalus* or *Candida pelliculosa* or *Hansenula anomala* or *Pichia anomala* case reports by PubMed service of the National Center for Biotechnology Information Search database.^1^ Cases including adult patients (age ≥ 18) with fungemia were included for further study.

## Results

### Clinical Characteristics of 13 Adult Patients Infected by *Wickerhamomyces anomalus*

During the study period, 2014–2016, a total of 13 adult patients with *W. anomalus* fungemia were identified, and their clinical manifestations are summarized in Table 1. In total, seven of them (53.8%) were female and six of them (46.2%) were male patients with the age range of 29–89 years (mean ± SD: 57.15 ± 17.22 years), among whom eight (61.5%) were classified as elderly (age ≥ 60 years). Of the 13 *W. anomalus* infected cases, 8 were collected from surgical ward (4 in general surgery, 2 in urological surgery, 1 in thoracic surgery, and 1 in cerebral surgery), 3 from intensive care unit (2 in thoracic surgery ICU and 1 in emergency ICU), and 2 from medical ward (1 in gastroenterology and 1 in cardiology) (Table 1). Given that some patients had more than one underlying conditions, solid malignancies (8/13; 61.5%), followed by hypertension (5/13; 39.5%), diabetes mellitus (4/13; 30.8%), and myocardial infarction (2/13; 15.4%), were the most prevalent complications (Table 1). The length of hospital stay ranged from 12 to 116 days (median: 55 days), and the time to first *W. anomalus* isolate was 4–50 days after admission (median: 23.3 days).

**TABLE 1:** Baseline characteristics of 13 adult patients infected by *W. anomalus*.

**Patients**	**P1**	**P2**	**P3**	**P4**	**P5**	**P6**	**P7**	**P8**	**P9**	**P10**	**P11**	**P12**	**P13**
Age/Sex	66/M	75/F	61/F	60/M	39/F	41/M	33/F	69/F	64/M	89/F	70/M	47/F	29/M
Ward	Thoracic Surgical ICU	Cerebral surgery	Thoracic Surgical ICU	General surgery	Gastroenterology	Emergency ICU	General surgery	General surgery	General surgery	Cardiology	Thoracic surgery	Urological surgery	Urological surgery
Underlying disease	MI, CKF, HTN, DM	Meningioma, intracranial infection, arrhythmia	Mitral valve prolapses	Vater ampulla carcinoma	Severe pancreatitis, HTN, pneumonia	Multiple trauma	Pancreatic cancer, DM	Gastric cancer, DM, HTN	Pancreatic cancer, HTN	MI, DM, CKF, HTN	Esophagus cancer, pulmonary infection	Renal cancer	Prostatic sarcoma
Hospital stays (day)	79	63	45	37	113	59	37	30	116	19	19	92	12
Time to first isolate (day)	50	45	23	24	12	26	25	23	17	16	17	21	4
Surgery	Yes	No	Yes	Yes	No	Yes	Yes	Yes	Yes	Yes	No	Yes	No
Prior mechanical ventilation	Yes	No	Yes	No	No	No	No	No	Yes	No	Yes	No	No
Prior antibiotic usage	Yes	Yes	Yes	Yes	Yes	Yes	Yes	Yes	Yes	Yes	Yes	Yes	Yes
Prior antifungal prophylaxis	No	Yes	No	No	No	Yes	No	No	No	No	Yes	No	No
Prior steroids	No	No	No	No	No	No	No	No	No	No	No	No	Yes
CVC/PICC	Yes	Yes	Yes	Yes	Yes	Yes	Yes	Yes	Yes	Yes	Yes	Yes	Yes
TPN	Yes	No	Yes	Yes	Yes	Yes	Yes	Yes	Yes	Yes	Yes	Yes	Yes
WBC (*10^9)	9.27	9.22	10.68	9.36	11.07	5.14	4.47	4.96	9.43	8.46	6.16	8.42	1.8
Neutrophil (*10^9)	5.97	6.03	7.85	8.04	8.95	3.72	3.61	4.39	6.98	6.75	5.76	7.24	1.2
T (°C)	40	38.4	36.5	38.7	38.7	39.1	38.9	38.5	39.8	39.2	38.2	39.4	39.5
MC	No	No	No	*Candida parapsilosis*	*Candida famata*	*Candida magnoliae*	No	No	No	No	No	*Candida glabrata*	No
Bacteremia	G+	G-	G-	No	G-	No	G-	G+	G+	No	No	G+	No
Antifungal therapy	VOR	FCZ/IZ	FCZ	FCZ	FCZ	VOR	FCZ	FCZ	FCZ + VOR	None	FCZ	ABLC	FCZ
Outcome	Died of septic shock	Died of multiple organ failure	Survival	Survival	Survival	Survival	Survival	Survival	Died of multiple organ failure	Died of multiple organ failure	Died of respiratory failure	Survival	Survival

*MI, myocardial infarction; HTN, hypertension; DM, diabetes mellitus; CKF, chronic renal failure; TPN, total parenteral nutrition; FCZ, fluconazole; VOR, voriconazole; IZ, itraconazole; ABLC, amphotericin B lipid complex; MC, mixed candidemia.*

Clinical manifestations were non-specific, and fever was the most common characteristic with the average being 38.8°C. All patients, except one (12/13; 92.3%), had temperatures over 38°C, and six patients (46.2%) presented repeated high fever (T > 39°C) despite the usage of antibiotics. Besides, we found that *W. anomalus* infections were always accompanied with infections caused by either other *Candida* species or bacteria. Mixed candidemia (MC) were detected in four patients (30.8%), namely, *C. parapsilosis*, *C. famata*, *C. magnoliae*, and *C. glabrata* (Table 1). Eight patients (61.5%) developed bacteremia during this period, with 50% Gram-negative and Gram-Positive bacteria equally found in blood samples (Table 1). In our study, only one patient (7.7%) had the white blood cell count lower than normal values (<4,000/mm^3^) and had mild neutropenia with blood neutrophil count being 1,200/mm^3^ during his episode of *W. anomalus* candidemia (Table 1).

### Risk Factors, Antifungal Treatment, and Outcomes

**FIGURE 1:**
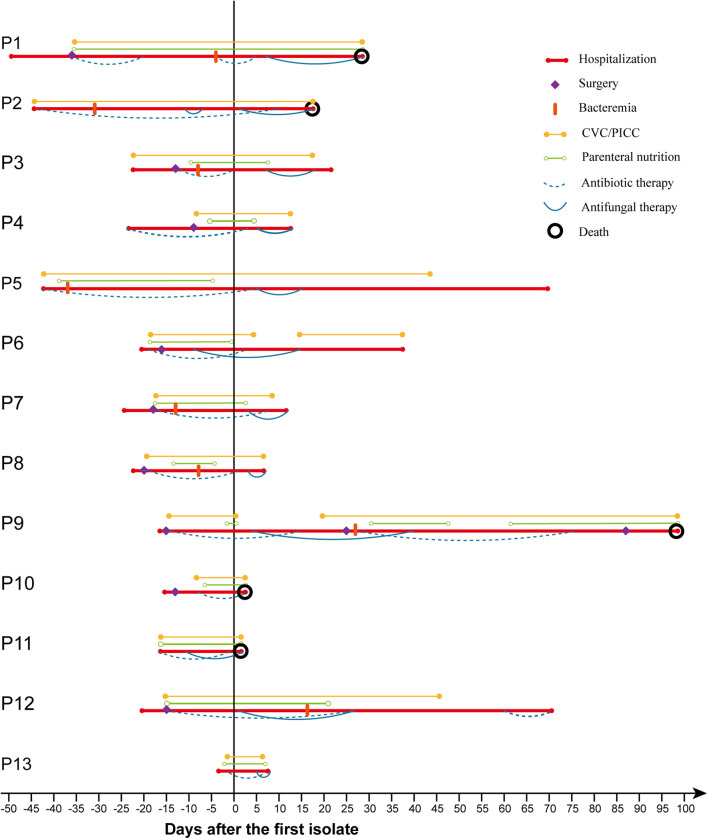
Clinical course, treatment, and outcomes of 13 cases of *W. anomalus* infections.

As shown in Figure 1 and Table 1, most patients had various risk factors for candidemia. Prior antibiotic usage (100%) and use of CVC/PICC (100%) were the predominant risk factors, followed by TPN (12/13; 92.3%), surgery (9/13; 69.2%), and prior mechanical ventilation (4/13; 30.8%). Eleven patients (84.6%) received antifungal therapy with triazole-based regimens including fluconazole (7/13; 53.8%), voriconazole (2/13; 15.4%), or combination of fluconazole with itraconazole (1/13; 7.7%) or voriconazole (1/13; 7.7%); one patient received amphotericin B lipid complex (ABLC); and one patient did not receive any antifungal treatment. Overall mortality rate was 38.5% (5/13), among whom four received triazoles and one was not treated.

### *In vitro* Susceptibility Testing

Overall, none of the isolates showed high MIC values against AMB (MIC range: ≤0.5–1 μg/ml; GM MIC: ≤0.5 μg/ml), while all isolates showed high MIC values against flucytosine (MIC range: 16–≥32 μg/ml; GM MIC: ≥32 μg/ml), which was significantly higher than the ECV reported for *C. albicans* (Pfaller and Diekema, 2012). Among the azoles tested, itraconazole had the highest MIC values (>0.125 μg/ml; 8/13, 61.5%), followed by voriconazole (>0.25 μg/ml; 3/13, 23%) and fluconazole (>4 μg/ml, 1/13, 7.6%). One isolate had NWT MIC to all azoles (#6), and two were NWT for voriconazole and itraconazole (#10 and #12) (Table 2). Before the first isolation of *W. anomalus*, patient #6 had received 9 days of prior voriconazole treatment due to the coinfection with *C. magnoliae*, while patients #10 and #12 did not receive any antifungal treatment before.

**TABLE 2:** Results of antifungal susceptibility of 13 *W. anomalus* strains.

**Patient**	**FC (μg/ml)**	**AMB (μg/ml)**	**FCZ (μg/ml)**	**IZ (μg/ml)**	**VOR (μg/ml)**
1	≥32	≤0.5	2	1	0.25
2	16	≤0.5	2	0.125	0.25
3	≥32	≤0.5	1	0.25	0.125
4	≥32	≤0.5	1	0.125	0.06
5	≥32	≤0.5	2	0.125	0.06
6	≥32	≤0.5	16	2	1
7	≥32	≤0.5	2	0.125	0.06
8	16	≤0.5	2	≤0.125	≤0.06
9	≥32	≤0.5	2	0.25	0.125
10	≥32	≤0.5	4	0.25	0.5
11	16	≤0.5	4	0.5	0.25
12	≥32	≤0.5	4	0.5	0.5
13	≥32	1	4	1	0.25
MIC range	16–≥32	≤0.5–1	1–16	≤0.125–2	0.06–1
GM MIC	≥32	≤0.5	2.6	0.31	0.18
MIC_50_	≥32	≤0.5	2	0.25	0.25
MIC_90_	≥32	≤0.5	4	1	0.5

*FC, flucytosine; AMB, amphotericin B; FCZ, fluconazole; IZ, itraconazole; VOR, voriconazole; MIC, minimum inhibitory concentration; GM, geometric mean.*

### Systemic Review of *Wickerhamomyces anomalus* Fungemia in Adult Patients

As shown in Tables 3, 4, we found 14 case reports including 15 adult patients (Milstoc and Siddiqui, 1986; Nohinek et al., 1987; Haron et al., 1988; Klein et al., 1988; Munoz et al., 1989; Salesa et al., 1991; Hirasaki et al., 1992; Neumeister et al., 1992; Goss et al., 1994; Kunova et al., 1996; Sumitomo et al., 1996; Kane et al., 2002; Chan et al., 2013; Mehta et al., 2020) and two outbreaks (Kalenic et al., 2001; Jung et al., 2018) totaling 15 adult patients with fungemia due to *W. anomalus* retrieved from the literatures. These adult patients (Table 3) ranged in age from 21 to 65 years, with the median being 44.7 years, which was much younger than our study (median: 57.2). Hematological (5/15; 33.3%) and solid malignancies (4/15; 26.7%) were the most common underlying disease. Fever was also the most common clinical manifestation, and three cases (20%) experienced neutropenia. From the literature, CVC (12/15; 80%) was the most common risk factors, and intravenous drug addiction was found among 13.3% of the cases (2/15; 13.3%). Most of these patients (9/15; 60%) were treated with AMB or ABLC, with 100% of those patients successfully treated. Notably, one patient died of *W. anomalus* infections despite receiving 200 mg fluconazole/day from day 19 of the clinical course, and this fungus continues to be isolated until the patients died at day 29 after admission to hospital for which the antifungal susceptibility testing was lacking (Neumeister et al., 1992).

**TABLE 3:** Summary of *W. anomalus* associated candidemia reported cases in adult patients from the literature.

**Study**	**Age/Sex**	**Underlying disease**	**Clinical manifestation**	**Neutropenia**	**Risk factors**	**Treatment**	**Outcome**
S1 (Salesa et al., 1991)	28/NA	AIDS, pulmonary tuberculosis	Fever (39°C)	No	Intravenous drug addiction	None	Cured
S2 (Neumeister et al., 1992)	65/M	Acute necrotizing pancreatitis	Fever	No	CVC, surgery	FCZ	Died
S3 (Chan et al., 2013)	21/M	Sickle cell disease	Fever, shaking chills	No	CVC	MF	Cured
S4 (Haron et al., 1988)	34/M	Acute myelocytic leukemia	Fever	Yes (1300)	CVC, chemotherapy	AMB	Cured
S5 (Hirasaki et al., 1992)	63/F	Advanced small cell lung cancer	Fever (39.9°C)	No	CVC, hyperalimentation, antibiotics, chemotherapy	FCZ	Cured
S6 (Kane et al., 2002)	32/M	Motor vehicle accident, renal failure	Fever (38.3°C)	No	CVC, surgery, antibiotics	ABLC	Cured
S7 (Goss et al., 1994)	22/NA	Chronic myeloid leukemia, bone marrow transplantation	Fever	No	CVC, hyperalimentation, antibiotics	AMB	Cured
S8 (Kunova et al., 1996)	46/F	Acute myeloid leukemia	Fever (38.5°C)	Yes (50)	Chemotherapy, CVC	FCZ/AMB	Cured
S9 (Milstoc and Siddiqui, 1986)	57/M	Multiple sclerosis	Fever	No	CVC	AMB	Cured
S10 (Nohinek et al., 1987)	40/M	Bicuspid aortic valve	Normal	No	Intravenous drug addiction	AMB	Cured
S11 (Sumitomo et al., 1996)	61/F	Colon carcinoma	Fever(40.6°C)	No	CVC	FCZ	Cured
S12 (Munoz et al., 1989)	51/F	Acute promyelocytic leukemia (M3)	High fever	No	CVC	AMB	Cured
S13 (Mehta et al., 2020)	36/M	B-cell acute lymphoblastic leukemia	Fever	Yes	Chemotherapy, CVC	FCZ	Cured
S14 (Klein et al., 1988)	59/F	Endometrial carcinoma	Fever	No	Surgery, parenteral nutrition	AMB	Cured
	55/F	Ovarian adenocarcinoma	Fever	No	CVC, parenteral nutrition, antibiotics	AMB	Cured

*M, male; F, female; NA, not available; None, no antifungal treatment; CVC, central venous catheter; AMB, amphotericin B; ABLC, amphotericin B lipid complex; FCZ, fluconazole; MF, micafungin.*

**TABLE 4:** Summary of *W. anomalus* associated candidemia reported by outbreaks in adult patients from the literature.

**References**	**No. of adult patients (age)**	**Ward**	**Main underlying disease**	**Risk factors**	**Treatment**	**Outcome**
Outbreak (Jung et al., 2018)	7 (18–78)	ICU (*n* = 5)	Polytrauma (*n* = 3); cancer (*n* = 3); diabetes and community-acquired pneumonia (*n* = 1)	CVC; mechanical ventilation; TPN; probable X-ray machine	Micafungin (*n* = 4); caspofungin (*n* = 1); fluconazole then micafungin (*n* = 2)	4/7 died
Outbreak (Kalenic et al., 2001)	8 (22–61)	ICU (*n* = 8)	Polytrauma (*n* = 4); cancer (*n* = 1); Crohn’s disease (*n* = 1); intestinal tuberculosis (*n* = 1); occlusion of a. iliaca communis (*n* = 1)	Duration of blood alkalosis	Fluconazole (*n* = 3); fluconazole + miconazole (*n* = 2)	3/8 died

*ICU, intensive care unit.*

Results obtained from the outbreaks (Table 4) indicted that infections caused by *W. anomalus* were prone to spread in ICU patients with multiple trauma (7/15) and cancers (4/15), and could cause high mortality in adult patients, with approximately half of these patients dying from infections (Kalenic et al., 2001; Jung et al., 2018). Despite of unestablished source of outbreak, the old cotton samples and probable X-ray machine were considered as the probable source of transmission of *W. anomalus* in these two outbreaks, and stopping the old cotton supplies use, disinfecting the X-ray cassettes with alcohol and quaternary ammonium compounds, as well as strict hand hygiene practices could prevent *W. anomalus* spread (Table 4). In the two outbreaks, fluconazole (3/15; 20%), caspofungin (1/15; 6.7%), miconazole (4/15; 26.7%), or combination of fluconazole with miconazole (4/15; 26.7%) were used as antifungal therapy, and eventually four adult patients (57.1%) in Jung’s study (Jung et al., 2018) and three (37.5%) in Kalenic’s study (Kalenic et al., 2001) died, with five patients receiving prior azole based treatments. When compared with the data obtained from sporadic reports, we can conclude that amphotericin B could be a suitable choice for the treatment of *W. anomalus* infections, and lipid complex amphotericin B can be used in patients with acute renal failure.

## Discussion

Numerous studies conducted have revealed the increasing incidence of infections caused by rare yeast species during the last decade. *Wickerhamomyces anomalus* is an emerging yeast species, and despite being associated with poor outcomes, therapeutic failure, and clonal outbreaks, our understanding about the clinical and microbiological characteristics of this species is limited. Therefore, the scope of the current study was to determine the clinical and microbiological characteristics of fungemia caused by *W. anomalus*, and to reach a deeper understanding, we performed a systematic literature review. Our study revealed that a relatively high mortality rate of 38.5% (5/13) and 36.4% (4/11) of the cases treated with azoles showed poor outcomes. Our literature review pointed to the efficacy of AMB formulations when compared to azoles. This is in agreement with our AFST data, where the vast majority of the isolates showed high MICs against azoles, while those for AMB were fairly low. Moreover, in line with other studies, we showed that CVC insertion, broad-spectrum antibiotic therapy, TPN, and surgery were the most notable risk factors, while neutropenia was not a prominent risk factor. Finally, our study highlights the importance of outbreaks due to *W. anomalus* among both adults and neonates and provided clues about effective therapeutic strategies.

In our study, we found most patients had various risk factors previously described for candidemia patients, including prior antibiotic usage, CVC/PICC, use of TPN, surgery, and mechanical ventilation (Ostrosky-Zeichner et al., 2007; Poissy et al., 2020). A 2018 publication revealed dwelling of CVC, TPN, usage of mechanical ventilation, and one medical staff were associated with the occurrence and transmission of *W. anomalus* fungemia (Jung et al., 2018). When compared to the other studies (Kalenic et al., 2001; Jung et al., 2018), herein, we found that solid tumors rather than multiple trauma or hematological malignancies were the most common underlying disease in adult patients. In addition, we found that *W. anomalus* infections were most commonly seen in older patients, especially those ≥60 years. Of note, our study showed that most of our cases had a mixed blood-borne infections of bacterial and *Candida* origin. Although some studies have reported a high rate of mixed bacterial/Candida bloodstream infections reaching 23% of total candidemia cases (Kim et al., 2013), the occurrence of MC is relatively low with incidence varying from 2 to 9.3%, and *C. albicans* is considered as the most common species in MC (Pappas et al., 2003; Mohd Tap et al., 2018). However, in our study, we found that approximately 61.5% *W. anomalus* cases were coinfected with bacteria, and 4 cases (30.8%) had polyfungal candidemia. Taken together, patients with preceding bacteremia, those with solid tumor, and elderlies potentially could be more prone to develop fungemia due to *W. anomalus*.

The literature data on the susceptibilities of *W. anomalus* are limited; previous studies including six isolates suggested fluconazole, AMB, and flucytosine showed optimal antifungal activity against *W. anomalus* (Kalenic et al., 2001). However, our *in vitro* antifungal susceptibility indicated that *W. anomalus* isolates have intrinsically high MIC values for flucytosine (≥16 μg/ml, 13/13, 100%) and also for azoles in general. Similar to our study, there are some reports indicating a low susceptibility to itraconazole (2 μg/ml; 8/8, 100%) (Lin et al., 2013) and decreased susceptibility to fluconazole (16.7%; resistant) (Pfaller et al., 2015) of *W. anomalus* isolates. In our study, however, none of the isolates showed high MICs for AMB with all strains MICs ≤1 μg/ml.

In our study, we found that prior treatment with azoles in some patients failed to control the deterioration of the disease. We speculate that fluconazole prophylaxis might be not suitable for *W. anomalus* infections, which is reflected by the observation of the high MIC values against azoles that may further reinforces the low efficacy of azoles among patients with fungemia due to *W. anomalus*. Azole antifungal agents were the main antifungal therapy in our study, but the prognosis was not satisfactory, and eventually four patients died despite being treated with azoles. Most clinicians prefer echinocandins as initial therapies for the management of invasive candidiasis (Pappas et al., 2018). Although some studies have reported the MICs of *W. anomalus* against echinocandins are relatively low, the prognosis of patients receiving echinocandin antifungal therapies does not seem satisfactory (Jung et al., 2018). As shown in Table 4, in Jung et al.’s study, although all seven adult patients received echinocandin therapies, four of them (4/7; 57.1%) died and three of these deaths were attributed to fungemia related to *W. anomalus* (Jung et al., 2018). Of note, an emerging body of evidence suggests that echinocandin-resistant non-*Candida albicans* species are a growing concern in clinical settings (Perlin, 2015; Kordalewska et al., 2018; Arastehfar et al., 2021). Regrettably, antifungal susceptibility testing of echinocandins was not performed in our center, and no patients were treated with echinocandins. Further studies are warranted to reach a definitive consensus regarding the utility of echinocandins for such patients. *In vivo* studies have suggested AMB as an efficacious alternative for patients with infections refractory to echinocandins or those infected with multidrug-resistant isolates (Olson et al., 2005; Binder et al., 2020). Moreover, clinical studies have suggested that LAMB is a safe and efficacious prophylactic option for invasive fungal infections in pediatric patients undergoing hematopoietic stem cell transplantation (Mendoza-Palomar et al., 2020). Systematic reviewing of the literature showed that amphotericin B seems to be the optimal choice for the treatment of *W. anomalus* infections, and lipid complex amphotericin B could be used in patients with acute renal failure (Goss et al., 1994; Kane et al., 2002). Since this fungus shows decreased susceptibility to several antifungal agents and can cause life-threatening and disseminated diseases in severely ill patients, antifungal treatment should be coupled with the results of antifungal susceptibility testing. However, the lack of ECVs and/or CBPs could be important considerations for clinical success, which stems from the limited number of fungemia cases due to *W. anomalus*. Altogether, these data indicate the low efficacy and poor outcomes associated with azoles, the high efficacy of AMB and its respective lipid derivatives, along with a dire need for antifungal susceptibility data of this species.

Although outbreak *W. anomalus* fungemia have been reported before, since this study was retrospective and these isolates were not preserved, we failed to assess the genetic similarity of such isolates, which is the main limitation of the current study. Nonetheless, it does not preclude the possibility of an ongoing outbreak with an unknown source of infection, which may require extensive environmental sampling and application of strict infection control strategies to curb this potential outbreak.

## Conclusion

In conclusion, *W. anomalus* has become an emerging pathogen, and for the first time, our study emphasizes that *W. anomalus* has recently emerged as a serious potential cause of candidemia in severe ill adult patients presented with various risk factors. In addition, considering the high MIC to flucytosine and triazoles, accurate species identification and treatment based on antifungal susceptibility testing could potentially result in a better clinical success. Our clinical data and systemic review suggested AMB as an effective antifungal agent, which was further supported by the low MIC values, while considering the high MICs, we do not advocate using flucytosine or itraconazole as the first choice to treat patients suffering from fungemia due to *W. anomalus*.

## Data Availability Statement

The original contributions presented in the study are included in the article/supplementary material, further inquiries can be directed to the corresponding authors.

## Author Contributions

JiZ, WF, and WP designed the study. JuZ, YD, and YX collected and analyzed the data. LZ and MX wrote the manuscript. AA, MI, and WL revised the manuscript. All authors have contributed to and approved the final manuscript.

## Conflict of Interest

The authors declare that the research was conducted in the absence of any commercial or financial relationships that could be construed as a potential conflict of interest.

## Publisher’s Note

All claims expressed in this article are solely those of the authors and do not necessarily represent those of their affiliated organizations, or those of the publisher, the editors and the reviewers. Any product that may be evaluated in this article, or claim that may be made by its manufacturer, is not guaranteed or endorsed by the publisher.

## Footnotes

1
https://www.ncbi.nlm.nih.gov/
